# B cell–derived exosomal tRNA-Pro-TGG served as a non-invasive biomarker and mediator of inflammation in progressive IgA nephropathy

**DOI:** 10.3389/fimmu.2025.1679290

**Published:** 2025-11-21

**Authors:** Heng Zhang, Guiyang He, Juanjuan He, Changyang Li, Lizhi Lv, Meng Jia, Fang Lu, Jun Liang, Junxiang Wu, Xinhao Zhou, Shaochang Jia, Ke Zen, Yanggang Yuan, Hongwei Liang

**Affiliations:** 1 Department of Emergency, Nanjing Drum Tower Hospital, School of Life Science and Technology, China Pharmaceutical University, Nanjing, China; 2 Jurong People’s Hospital, Jiangsu, China; 3 Department of Rheumatology and Immunology, Nanjing Drum Tower Hospital, Medical School of Nanjing University, Nanjing, China; 4 University of Edinburgh, Edinburgh, United Kingdom; 5 Department of Oncology, Nanjing Jinling Hospital, Affiliated Hospital of Medical School, Nanjing University, Nanjing, China; 6 Department of Nephrology, The First Affiliated Hospital of Nanjing Medical University, Nanjing, China

**Keywords:** IgA nephropathy, exosomes, tRNA-Pro-TGG, biomarkers, solubleTNFR1, B lymphocytes, inflammation

## Abstract

IgA nephropathy (IgAN) is the most common form of primary glomerulonephritis and a leading cause of end-stage renal disease globally. Although mesangial IgA deposition defines its pathology, this alone does not predict disease progression. Current biomarkers lack specificity for forecasting outcomes or guiding early intervention. Recent advances have highlighted the potential of exosome-derived tRNA-derived small RNAs (tsRNAs) as novel diagnostic tools and mediators of disease processes, but their role in IgAN remains insufficiently explored. In this study, serum exosomes were isolated from patients with progressive or non-progressive IgAN and healthy controls. tsRNA expression profiles were obtained using small RNA sequencing and validated by qRT-PCR. Bioinformatic analyses were conducted to identify target pathways. Functional effects of candidate tsRNAs were evaluated using luciferase reporter assays, tsRNA mimic/antago transfections, and co-culture of B cell-derived exosomes with collecting duct epithelial cells (CDECs). Among 566 identified exosomal tsRNAs, tRNA-Pro-TGG was significantly upregulated in patients with progressive IgAN. It was enriched in B lymphocytes and correlated with serum soluble TNFR1 levels. Functional assays revealed that exosomal tRNA-Pro-TGG suppressed MAPK translation and activated proinflammatory responses in CDECs, including increased secretion of TNF-α, IL-6, and CCL2. ROC analysis demonstrated its robust diagnostic power for distinguishing progressive from non-progressive disease (AUC = 0.9618). This study identifies exosomal tRNA-Pro-TGG as a novel, non-invasive biomarker for IgAN progression and implicates it as a mediator of immune-driven renal inflammation. These findings offer valuable insights into IgAN pathogenesis and support the potential clinical utility of tsRNA-based diagnostics in nephrology.

## Introduction

IgA nephropathy (IgAN), the most prevalent primary glomerulonephritis worldwide, poses a considerable lifetime risk of kidney failure (1). Its global burden has risen from 8.8 million cases in 2015 to 9.3 million in 2020, with China accounting for nearly one-quarter of cases; by 2030, prevalence is projected to reach 10.2 million globally and 2.4 million in China (2). Typically presenting in young adults, IgAN is often identified through asymptomatic hematuria and variable proteinuria, with individuals aged 20–40 comprising the majority of cases (2). While mesangial IgA deposition is the histological hallmark, its severity does not predict long-term renal outcomes, suggesting that other factors contribute to progressive glomerular and tubulointerstitial damage (3).

Liquid biopsy offers a non-invasive approach to detect molecular signatures of disease through analysis of body fluids. Among emerging biomarkers, tRNA-derived small RNAs (tsRNAs)-a subclass of small non-coding RNAs approximately 30 nucleotides in length-have gained attention for their regulatory roles in cellular processes. Classified into 5′ tsRNAs, inner tsRNAs, 3′ tsRNAs, and 3′CCA tsRNAs based on their origin from precursor or mature tRNAs (4, 5), these molecules influence cell proliferation, translation, mRNA silencing, apoptosis, and epigenetic modulation through diverse mechanisms including ribosome biogenesis and miRNA-like regulation (5, 6). For instance, 5′tiRNA-Pro-TGG may facilitate the progression of serrated pathway colorectal cancer (CRC) by modulating metabolic and immune pathways through its interaction with HPSE2 and regulation of HPSE2 expression in sessile serrated lesions (SSLs) and BRAF-mutant CRC (7, 8). Recent work by Luo et al. identified 143 upregulated and 202 downregulated tsRNAs in peripheral blood mononuclear cells from patients with IgAN, suggesting their potential involvement in disease pathogenesis (9). Notably, tsRNAs are enriched in exosomes-membrane-bound extracellular vesicles found in multiple body fluids-which range from 30 to 150 nm in size and have emerged as promising diagnostic biomarkers (10). However, the expression patterns and diagnostic relevance of exosomal tsRNAs in IgAN remain unclear and warrant further investigation.

In our study, we observed significant dysregulation of exosomal tsRNAs in IgA nephropathy (IgAN) compared to healthy controls. Notably, exosomal tsRNA derived from tRNA-Pro-TGG was markedly elevated in serum exosomes from IgAN patients and could differentiate between disease progression and non-progression. Further analysis suggests that these tsRNAs are likely sourced from activated B cells and may contribute to disease progression by exerting a proinflammatory effect on the distal nephron, specifically inducing phenotypic changes in collecting duct epithelial cells (CDECs).

## Methods

### Ethical approval and sample collection

Blood samples were collected from patients with biopsy-confirmed IgA nephropathy (IgAN) and healthy controls (HC) at Nanjing Drum Tower Hospital, Jurong People’s Hospital, and the First Affiliated Hospital of Nanjing Medical University (**Supplementary Table S1**). All IgAN patients were aged over 18 years and had not received immunomodulatory treatment for at least 12 months prior to blood collection. Patients were classified as having progressive IgAN (IgANp) if they experienced a >100% increase in serum creatinine or developed kidney failure within 5 years, and as nonprogressive (IgANnp) if serum creatinine changes were <10% during the same period. All participants provided written informed consent, and the study protocol was approved by the Ethics Committees of all participating institutions.

### Exosome extraction

Exosomes were extracted from 100µL serum using WGA-conjugated magnetic beads as previously described (11–15). For exosome characterization, serum was centrifuged at 3000×g for 10 minutes, filtered through 0.45 µm membranes (Millipore, HAWP04700), and incubated with WGA-conjugated magnetic beads at room temperature for 1 hour. Bead-bound exosomes were separated using a magnetic rack, washed twice with phosphate-buffered saline (PBS), and eluted with 500 mM N-acetyl-D-glucosamine (GlcNAc) in PBS. The beads were washed (20 µL beads with 400 µL PBS) and incubated with 400 µL GlcNAc buffer for 15 minutes before magnetic separation and eluate collection for downstream analyses. Western blotting was performed with antibodies against CD63, TSG101, ALIX, and Calnexin, and HRP-conjugated secondary antibodies (Abcam) for detection. For RNA sequencing and Quantitative real-time reverse-transcription PCR (qRT-PCR), serum was processed similarly but centrifuged at 3000×g for 20 minutes at 4 °C before filtration. The resulting filtrate was incubated with WGA-conjugated beads for 2 hours, and bead-exosome complexes were isolated, washed twice with PBS, and subjected to RNA extraction using TRIzol reagent (Invitrogen).

### Sequence processing of tRFs and tiRNAs

The purity and concentration of total RNA samples were assessed using the NanoDrop 8000 UV-Vis spectrophotometer (NanoDrop Technologies) and Agilent 4200 TapeStation (Agilent Technologies). tRF and tiRNA-seq libraries were prepared using the rtStar™ tRF and tiRNA Pretreatment Kit (Arraystar Inc.), which removed RNA modifications that could interfere with small RNA sequencing, including 3′-aminoacyl deacylation, 3′-cP removal, 5′-OH phosphorylation, and m1A/m3C demethylation. RNA was then ligated to 3′ and 5′ small RNA adapters, followed by cDNA synthesis and amplification using Illumina primers. Approximately 134–160 bp PCR-amplified fragments were purified via PAGE gel, and library quality was assessed using the Agilent BioAnalyzer 2100. Libraries were denatured, diluted to 1.8 pM, and loaded onto the Illumina NextSeq 500 system for sequencing with the NextSeq 500/550 V2 kit (Illumina). tsRNAs were identified through a well-established bioinformatic workflow. Briefly, the raw sequencing data generated by the Illumina NextSeq 500 platform were processed by removing adapter sequences and low-quality reads. The cleaned reads were then aligned to mature and precursor tRNA reference sequences obtained from the GtRNAdb (Genomic tRNA Database) and tRNAscan-SE annotated human tRNA datasets, using Bowtie with parameters allowing for small RNA alignment. The aligned reads were quantified and normalized to transcripts per million (TPM). The expression profiles of tRFs and tiRNAs were subsequently analyzed using the edgeR package in R for differential expression analysis (FDR cutoff of 0.05). Principal component analysis (PCA), hierarchical clustering, and volcano plots were generated using R packages including FactoMineR, factoextra, pheatmap, and ggplot2. The potential mRNA targets of differentially expressed tsRNAs were predicted using TargetScan and miRDB, based on sequence complementarity and binding site conservation. The resulting gene lists were then subjected to GO and KEGG pathway enrichment analysis using the PANTHER database, to infer potential biological processes and signaling pathways indirectly regulated by tsRNAs.

### Quantitative real-time reverse-transcription PCR

The purity and concentration of total RNA samples were assessed using the NanoDrop 8000 UV-Vis spectrophotometer (NanoDrop Technologies). tiRNAs were reverse-transcribed into cDNA using a Bulge-Loop miRNA qRT-PCR Starter Kit (Ribobio, Guangzhou, China). Subsequently, qPCR was performed with SYBR Premix Ex Taq (Takara), as instructed by the manufacturer. The primers of qPCR were list in **Supplementary Table S2**. To calculate the expression levels of the TRNA-Pro-TGG, a series of synthetic TRNA-Pro-TGG oligonucleotides (Ribobio, Guangzhou, China) at known concentrations in water were also reverse-transcribed and amplified. The quantity of TRNA-Pro-TGG in the cell was then calculated by referring to the standard curve.

### Analysis of RNA protection from RNase

Exosomes isolated using WGA-conjugated magnetic beads were treated with RNase (5µg/mL) and incubated at 37°C for 60 minutes. Subsequently, RNA was extracted using TRIzol reagent (Invitrogen) and analyzed by qRT-PCR. Synthetic tRNA-Pro-TGG subjected to the same RNase treatment and incubation conditions served as a positive control.

### Quantitative sTNFR1

sTNFR1 were measured in samples using Human TNFR1 (Soluble-60 kDa) ELISA Kit (Invitrogen) as the instructions.

### Cell culture

To prepare fetal bovine serum (FBS) for cell culture applications, extracellular vesicles (EVs) were depleted via a strict ultracentrifugation procedure. This process first involved centrifugation at 3,000 × g for a 30-minute duration, after which the serum was filtered using 0.45 μm filters (Millipore, catalog number HAWP04700). Subsequent steps included a 30-minute centrifugation at 10,000 × g, followed by ultracentrifugation conducted at 120,000 × g for 70 minutes. Such a stepwise treatment was critical to achieving thorough elimination of remaining EVs before the FBS was utilized in cell culture experiments. Human collecting duct epithelial cells (CDECs) were cultured in Dulbecco’s modified Eagle’s medium-Ham’s F:12 (DMEM/F-12), supplemented with ITS solution (5μg/ml insulin, 5 μg/ml transferrin 5μg/ml, and 30nM selenium; Thermo Fisher Scientific), 2 mM glutamine (Life Technologies), 50 nM dexamethasone (Sigma Aldrich), 2% EVs removed fetal calf serum, and 1% penicillin/streptomycin. Conditionally immortalized human glomerular mesangial cells (CIHGM-1) were maintained in RPMI1640 medium (16).

To investigate the role of TRNA-Pro-TGG, CDECs were plated in 12-well plates at a density of 10 (5) cells/ml in complete medium. After removal of the growth medium, cells were cultured in 0.5 ml serum-free medium, followed by transfection with tsRNA mimic using Lipofectamine™ RNAiMAX Transfection Reagent (Invitrogen), as per the manufacturer’s protocol.

To investigate the role of exosomes derived from B cells, CDECs were plated in 12-well plates at a density of 10 (5) cells/ml in complete medium. After removal of the growth medium, cells were cultured in 0.5 ml serum-free medium, followed by transfection with tsRNA antago using Lipofectamine™ RNAiMAX Transfection Reagent (Invitrogen), as per the manufacturer’s protocol. After 24 hours, EVs derived from sTNFR1 treated B cell medium was added for an additional 24 hours. Supernatants were collected, and RNA was extracted from the CDECs using TRIzol (Applied Biosystems).

### Cell sorting and sTNFR1 analyses

B cells and T cells was sorted by the CD19 MicroBeads or CD3 MicroBeads (Miltenyi Biotec, USA). B cells were maintained in R5 medium, consisting of RPMI 1640 supplemented with 5% human serum (Sigma-Aldrich), 55 µM 2-mercaptoethanol, 2 mM L-glutamine, 100 U/mL penicillin, 100 µg/mL streptomycin, 10 mM HEPES, 1 mM sodium pyruvate, and 1% MEM nonessential amino acids (all from Invitrogen). T cells were cultured in complete RPMI medium (500 mL total volume) containing 439.5 mL RPMI 1640 with GlutaMAX™ I supplement and 25 mM HEPES (Gibco, Life Technologies, Paisley, UK), 5 mL penicillin–streptomycin (10,000 U/mL penicillin and 10,000 µg/mL streptomycin; Gibco), 5 mL MEM sodium pyruvate (100 mM; Gibco), 0.5 mL 2-mercaptoethanol (1000×; Gibco), and 50 mL heat-inactivated human serum. To investigate the role of sTNFR1 on the B cells of IgAN patients, the B cells of IgAN patients were treated with recombinant soluble tumor necrosis factor receptor 1 (sTNFR1; sourced from Insight Biotechnology) at a concentration of 12.5 ng/ml.

### Statistics

Sequencing data were quantified as transcripts per million (TPM), calculated by dividing the number of reads for each tsRNA by the total mapped reads and multiplying by 1 million. tsRNAs with a mean group read count ≥ 10 TPM were selected for analysis. Relative tsRNA expression levels were determined using the Livak equation. Statistical analyses were performed using GraphPad Prism 9. Normality was assessed with the D’Agostino-Pearson test. Comparisons between two variables were made using the Student’s t-test (parametric) or Mann-Whitney test (nonparametric), as appropriate. For multiple comparisons, a one-way ANOVA with Bonferroni *post hoc* correction (parametric) or a Kruskal-Wallis test with Tukey’s *post hoc* test (nonparametric) was applied. A 95% confidence interval and a significance threshold of P ≤ 0.05 were used for all tests.

## Results

### Characterization of exosomes

**Figure 1:**
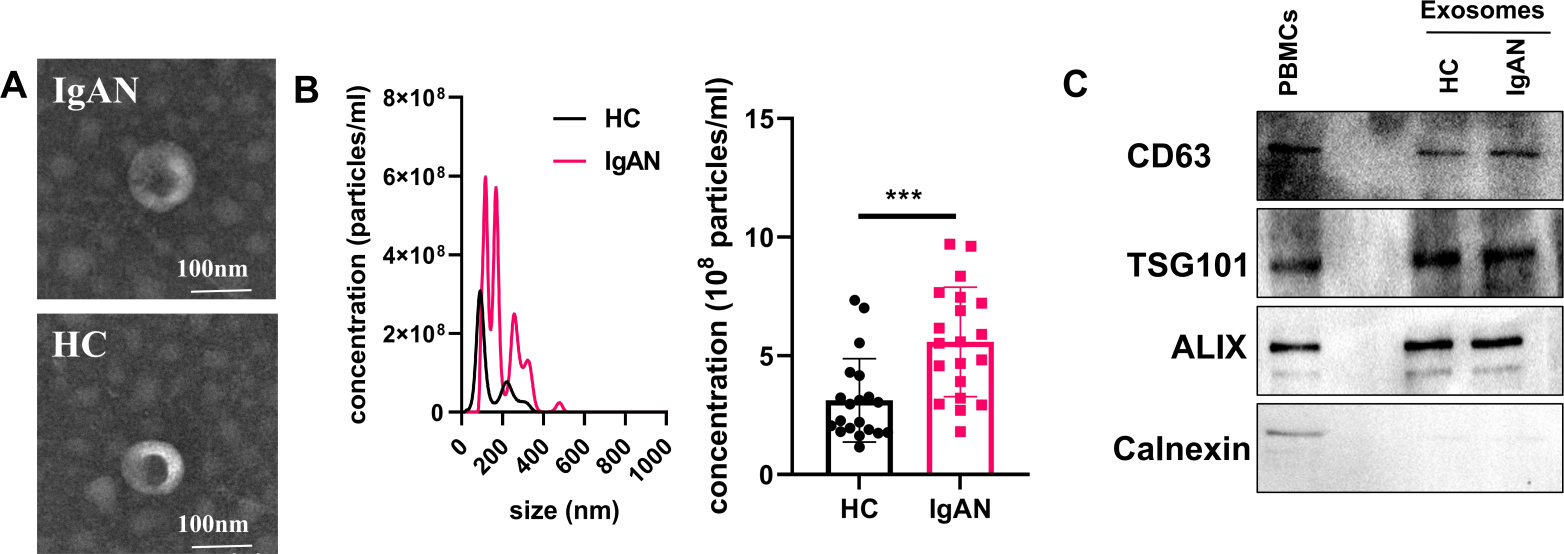
Isolation and characterization of exosomes. **(A)** TEM analysis of exosomes. The scale bar represents 100 nm. **(B)** NTA quantification of exosomes. *p* values are from unpaired Student’s t test. **(C)** Western blot analysis of exosomes. The data from three independent experiments (n = 3) are presented as the means ± SDs. ns: p > 0.05; ***p < 0.001.

Serum exosome morphology was assessed by TEM, and particle size was measured using NTA. The exosomes appeared elliptical or bowl-shaped, with an average diameter of 100 nm (**Figures 1A, B**). Consistent with previous report (17), the number of exosomes was significantly higher in IgAN patients compared to healthy controls (**Figure 1B**). Western blot analysis confirmed the presence of typical EV markers, including CD63,TSG101 and ALIX, and the absence of calnexin (**Figure 1C**).

### Serum exosomal tsRNAs are dysregulated in IgA nephropathy

**Figure 2:**
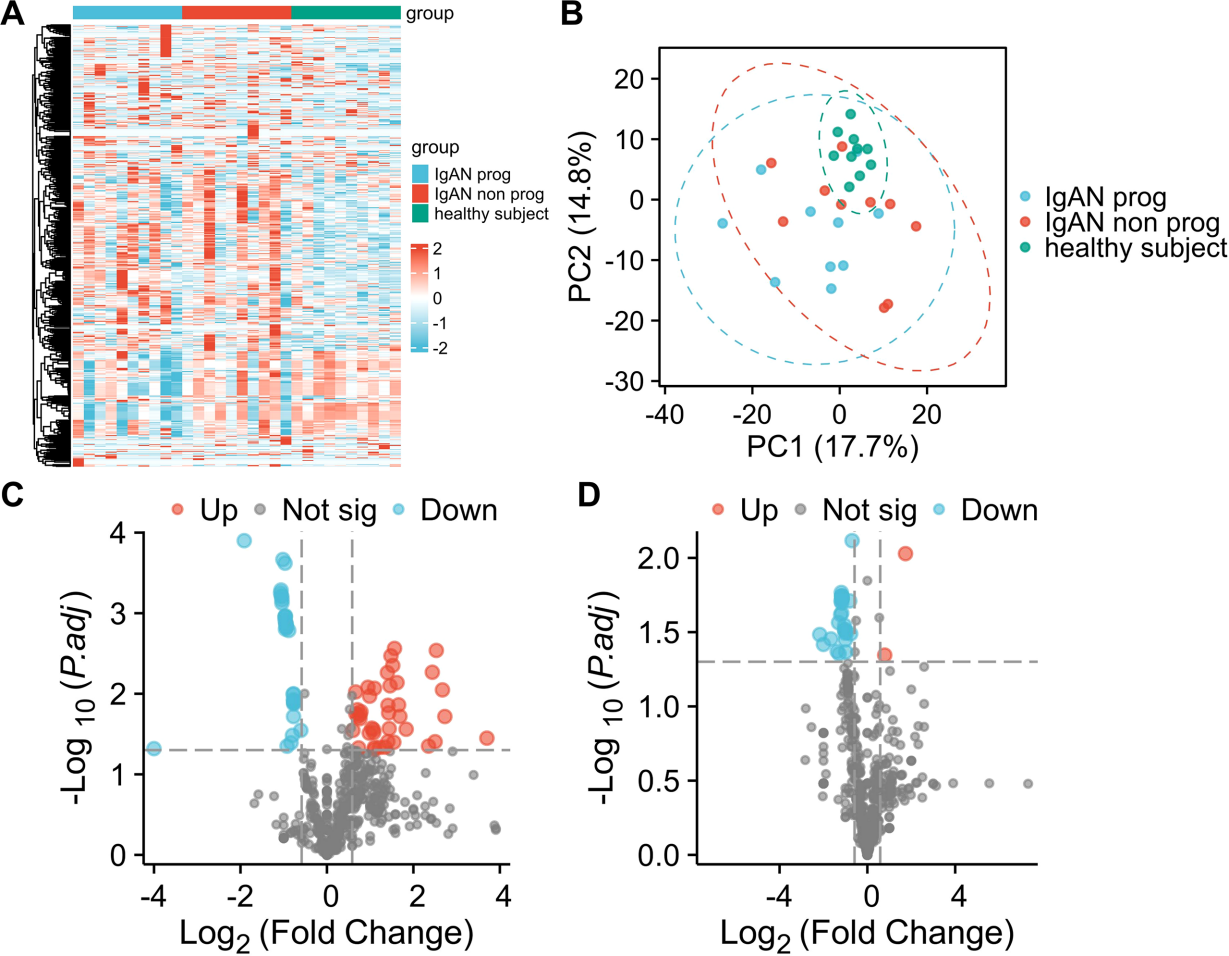
Heatmap **(A)**, PCA **(B)** and volcano plot of progressive IgAN (IgAN prog) vs HC **(C)** and progressive IgAN (IgAN prog) vs nonprogressive IgAN (IgAN non prog) **(D)** analysis of tsRNA sequencing data of serum exosomes from IgAN patients and Healthy controls (Healthy subject).

To profile tsRNAs in IgAN patients, participants were categorized into three groups: IgAN with progressive disease (IgAN prog), nonprogressive disease (IgAN non prog), and healthy controls (HC). Exosomes were extracted from serum samples of 10 IgAN prog, 10 IgAN non prog, and 10 HC participants, followed by small RNA sequencing. A total of 566 distinct tsRNAs were identified, with an average of 250 million counts per sample (**Supplementary Table S3**). A heatmap based on stratified clustering of tsRNAs and participant groups demonstrated consistent expression trends within each group (**Figure 2A**). Principal component analysis (PCA) further distinguished serum exosomal tsRNAs between IgAN patients and HCs (**Figure 2B**). Differential expression analysis revealed 77 tsRNAs with a fold change ≥1.5 and *p*-value <0.05 between IgAN and HC, including 43 upregulated and 34 downregulated tsRNAs (**Figure 2C**, **Supplementary Table S3**). In comparison between IgAN prog and IgAN non prog, 23 tsRNAs were differentially expressed, with 3 upregulated and 20 downregulated (**Figure 2D**, **Supplementary Table S3**).

### Gene ontology and Kyoto encyclopedia of genes and genomes enrichment analyses of the dysregulated tsRNAs

**Figure 3:**
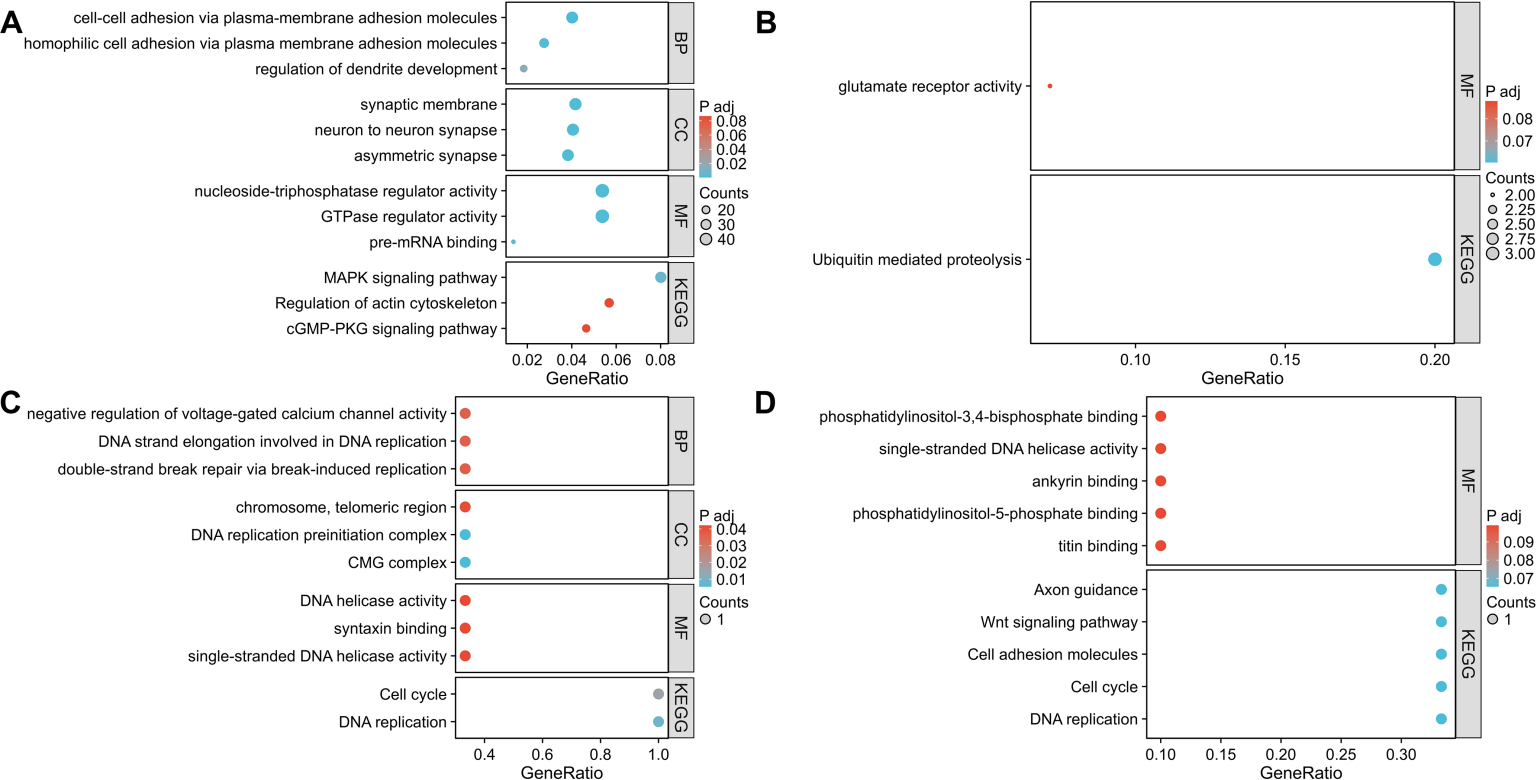
GO and KEGG analyses of upregulated tsRNAs and downregulated tsRNAs of progressive IgAN vs HC **(A, B)** and progressive IgAN vs nonprogressive IgAN **(C, D)**.

TargetScan and miRDB was used to predicted the potential mRNA targets of differentially expressed tsRNAs based on sequence complementarity and binding site conservation (**Supplementary Tables S4**, **S5**). The resulting gene lists were then subjected to GO and KEGG pathway enrichment analysis using the PANTHER database, to infer potential biological processes and signaling pathways indirectly regulated by tsRNAs. GO and KEGG pathway analyses were performed to investigate the functional roles of dysregulated tsRNAs in serum exosomes, using the PANTHER database. Upregulated tsRNAs in exosomes from IgAN were primarily associated with GTPase regulator activity, nucleoside-triphosphatase regulation, the MAPK signaling pathway, and the cGMP-PKG pathway (**Figure 3A**). Conversely, downregulated tsRNAs were linked to glutamate receptor activity and ubiquitin-mediated proteolysis (**Figure 3B**). In IgAN prog, upregulated tsRNAs were primarily involved in the negative regulation of voltage-gated calcium channels, DNA replication, and cell cycle progression (**Figure 3C**), while downregulated tsRNAs were associated with DNA replication, cell cycle, and Wnt signaling (**Figure 3D**). These results suggest a role for differentially expressed tsRNAs in the inflammatory processes driving IgAN progression.

### Serum exosomal tRNA-Pro-TGG serve as biomarker for IgA nephropathy diagnosis

TRNA-Pro-TGG was significantly elevated in both IgAN prog compared to IgAN non prog and in IgAN compared to HCs (**Supplementary Table S3**). RNA sequencing analysis revealed an area under the ROC curve of 0.81 for distinguishing IgAN prog from IgAN non prog, and 0.805 for distinguishing IgAN from HCs (**Supplementary Figures S1A, B**). Based on these findings, tRNA-Pro-TGG was selected for further investigation. To further confirm that tRNA-Pro-TGG was encapsulated within exosomes, exosomes were treated with RNase. Exosomal tRNA-Pro-TGG remained intact after RNase treatment, whereas free tRNA-Pro-TGG was rapidly degraded (**Supplementary Figure S1C**).

**Figure 4:**
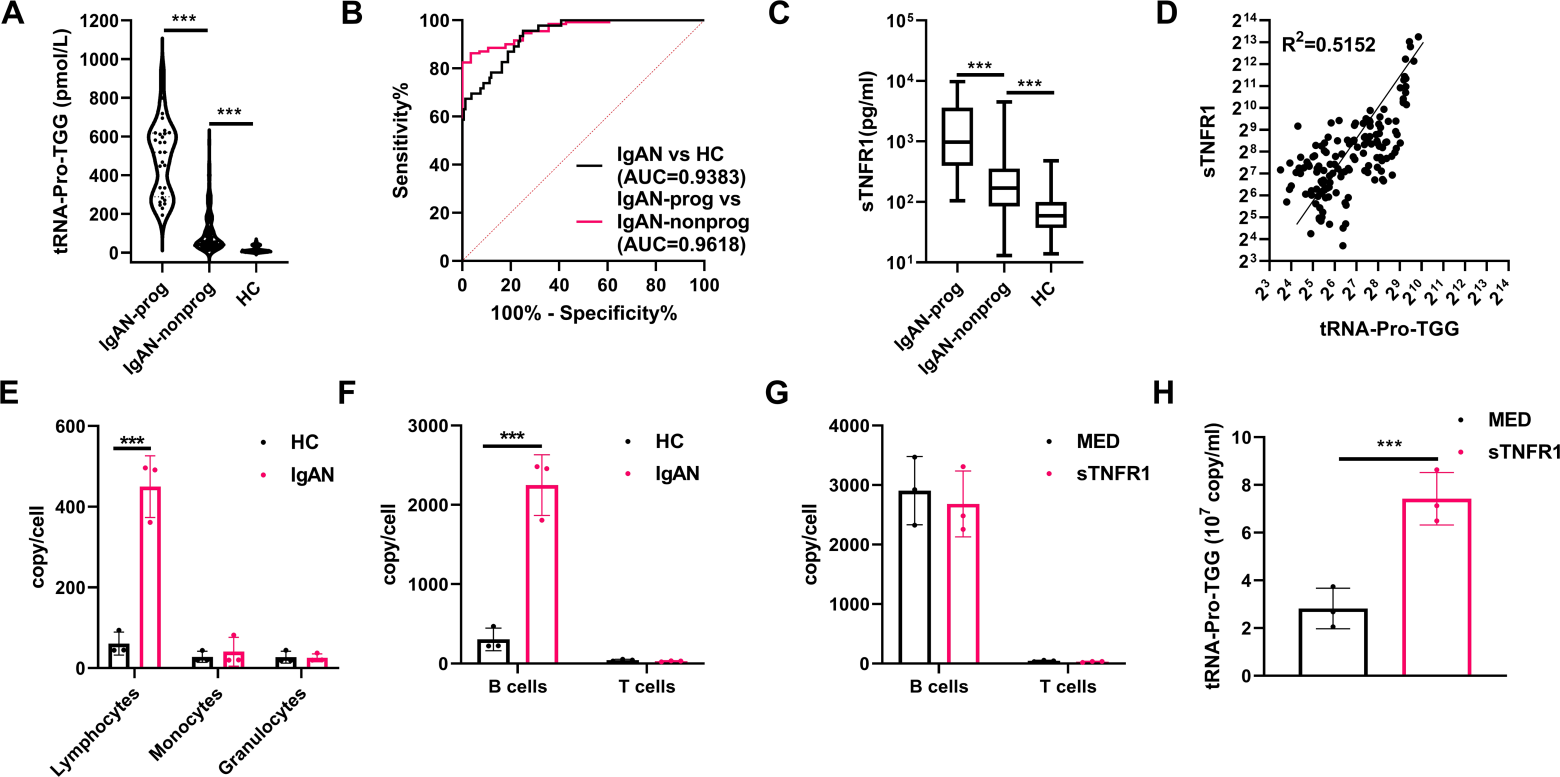
TsRNAs contained in serum exosomes used as biomarkers for diagnosing IgAN. **(A)** tRNA-Pro-TGG in serum exosomes from IgAN patients and Healthy controls. **(B)** ROC curve analysis of the tRNA-Pro-TGG in serum exosomes to distinguish IgAN from HC, and IgAN prog from IgAN non prog. **(C)** sTNFR1 in serum exosomes from IgAN patients and Healthy controls. **(D)** serum exosome tRNA-Pro-TGG content correlates with extent of serum sTNFR1 in IgA nephropathy (IgAN). **(E)** tRNA-Pro-TGG measured by RT-qPCR in lymphocyte, monocyte, and granulocyte subsets in healthy subjects (HC) and IgAN. **(F)** tRNA-Pro-TGG measured by RT-qPCR in B cells and T cells in healthy subjects (HC) and IgAN. **(G)** B cells from IgAN patients were exposed to sTNFR1 (12.5 ng/ml) or medium only (MED) for 4 hours and tRNA-Pro-TGG measured by RT-qPCR. **(H)** Exosomes of B cells from IgAN patients were exposed to sTNFR1 (12.5 ng/ml) or medium only (MED) for 4 hours and tRNA-Pro-TGG measured by RT-qPCR. The data from three independent experiments (n = 3) are presented as the means ± SDs. *p* values are from unpaired one-way ANOVA. ns: p > 0.05; ***p < 0.001.

To evaluate the diagnostic potential of tRNA-Pro-TGG in serum exosomes for IgAN, its expression was assessed in a validation cohort of 50 IgAN patients with progressive disease (IgAN prog), 50 without progressive disease (IgAN non prog), and 50 healthy controls (HC) using RT-qPCR (**Supplementary Table S1**). The results showed significant upregulation of tRNA-Pro-TGG in serum exosomes from IgAN prog compared to IgAN non prog, and in IgAN compared to HC (**Figure 4A**). ROC curve analysis demonstrated that tRNA-Pro-TGG effectively differentiated IgAN prog from IgAN non prog (AUC = 0.9618, sensitivity 95.65%, specificity 84.84%) and IgAN from HC (AUC = 0.9383, sensitivity 96.43%, specificity 86.26%) (**Figure 4B**).

### Exosomal tRNA-Pro-TGG content correlate with sTNFR1 and tRNA-Pro-TGG is enriched in B lymphocytes in IgA nephropathy

Previous studies have demonstrated that elevated serum levels of sTNFR1 correlate with the severity of various kidney diseases, including interstitial fibrosis in IgAN (17). Our results further confirm a significant increase in sTNFR1 levels in IgAN patients (**Figure 4C**). We also observed a strong correlation between the exosomal content of tRNA-Pro-TGG and sTNFR1 levels (**Figure 4D**), suggesting that lymphocytes are likely the source of tRNA-enriched exosomes. In line with this, tRNA-Pro-TGG was predominantly expressed in lymphocytes (**Figure 4E**). To investigate whether its expression was specific to B or T cell subsets, we enriched peripheral blood B and T cells using magnetic beads targeting CD19 or CD3. qRT-PCR analysis revealed that tRNA-Pro-TGG was primarily enriched in B cells (**Figure 4F**). Exosomes of B cells were extracted from the culture medium of B cells using WGA-conjugated magnetic beads as previously described (11–15), and characterized by TEM and western blotting (**Supplementary Figure S3**). Furthermore, consistent with prior findings that sTNFR1 modulates miR-483-5p content in B cell-derived exosomes (17), we observed that sTNFR1 regulates the release of tRNA-Pro-TGG-enriched exosomes from B cells in IgAN patients, without affecting intracellular tRNA-Pro-TGG expression (**Figures 4G, H**).

### B cell–derived tRNA-Pro-TGG-containing exosomes activate collecting duct epithelial cells

**Figure 5:**
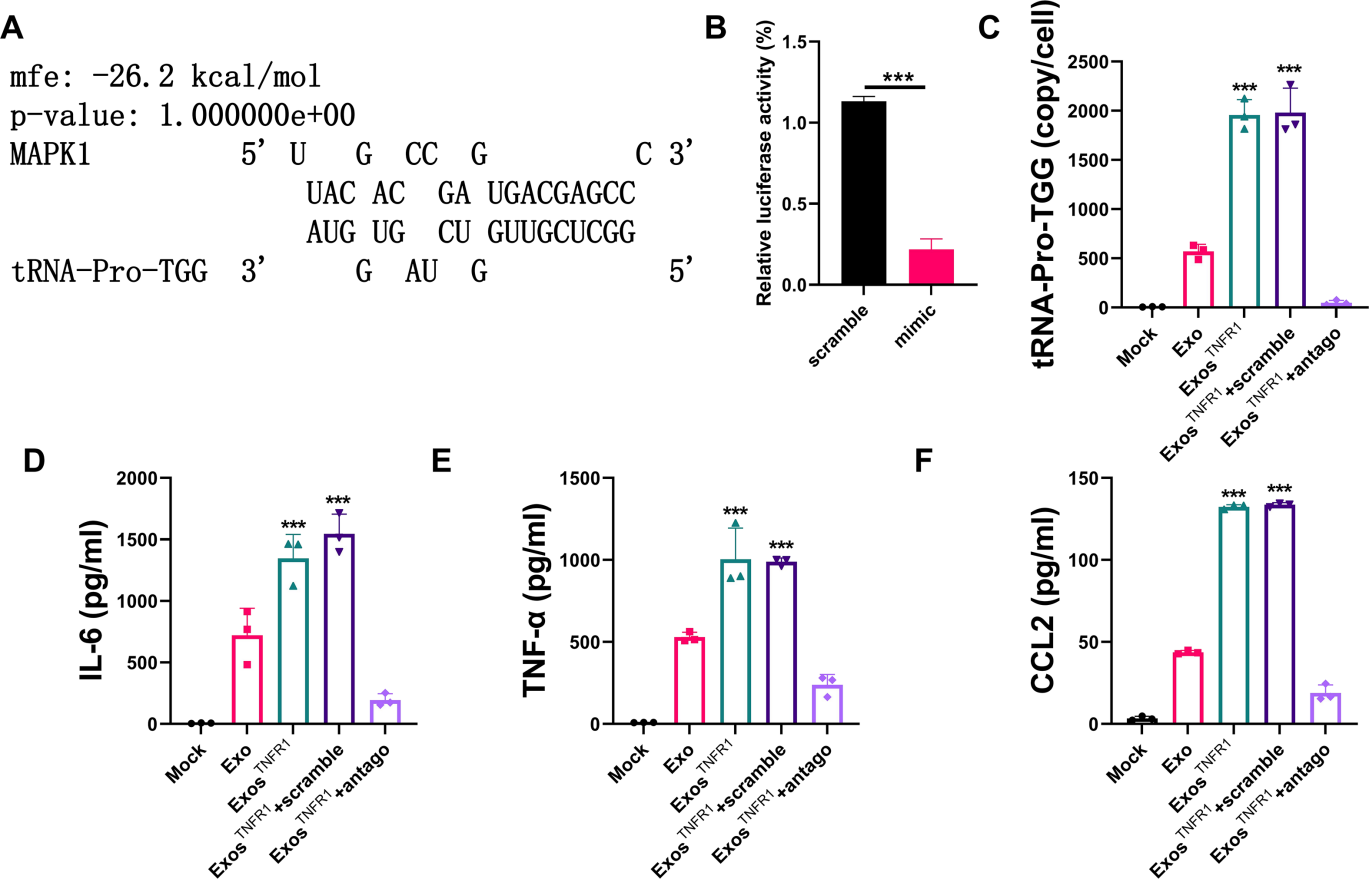
B cell derived exosome induction of a proinflammatory phenotype in collecting duct epithelial cells is tRNA-Pro-TGG dependent. **(A)** Graphical representation of the predicted duplex formed by the MAPK and tRNA-Pro-TGG. **(B)** Analysis of the luciferase reporter assay. Firefly luciferase reporters containing tRNA-Pro-TGG binding sites were cotransfected into 293T cells with equal doses of the tRNA-Pro-TGG mimic and scramble RNA. **(C)** The changes of tRNA-Pro-TGG in CDECs. **(D-F)** The pattern of changes in IL-6, TNF-αand CCL2. The data from three independent experiments (n = 3) are presented as the means ± SDs. *p* values are from unpaired one-way ANOVA. ns: p > 0.05; ***p < 0.001.

Bioinformatics analyses using TargetScan (18) and miRdb (19) identified several enriched KEGG pathways, including the MAPK and PI3K-Akt pathways, as potential targets modulated by tRNA-Pro-TGG (**Supplementary Figure S2**). MAPK emerged as a key protein in these pathways. The predicted interaction between tRNA-Pro-TGG and MAPK binding sites is shown in **Figure 5A**. To confirm whether tRNA-Pro-TGG directly targets MAPK, we fused the full-length MAPK gene, containing the presumed binding sites, downstream of the firefly luciferase gene in a reporter plasmid. This plasmid was co-transfected into 293T cells with a β-gal control plasmid and tRNA-Pro-TGG mimic (or scrambled control RNA). Transfection with the tRNA-Pro-TGG mimic resulted in increased tRNA-Pro-TGG content (**Supplementary Figure S4A**). As expected, luciferase activity was significantly lower in 293T cells transfected with the tRNA-Pro-TGG mimic (**Figure 5B**).

To assess the effects of tRNA-Pro-TGG enriched exosomes derived from peripheral B lymphocytes on renal cells, exosomes isolated from sTNFR1-treated B cells were co-cultured with a human collecting duct epithelial cell line (CDEC) or a human mesangial cell line (CIHGM-1). Treatment with exosomes from sTNFR1 stimulated B cells markedly increased tRNA-Pro-TGG expression in CDECs but not in CIHGM-1 cells (**Figure 5C**, **Supplementary Figure S4B**). Furthermore, transfection of CDECs with a tRNA-Pro-TGG mimic enhanced intracellular tRNA-Pro-TGG levels and subsequently suppressed MAPK protein expression (**Supplementary Figures S4D–E**), indicating that tRNA-Pro-TGG directly inhibits MAPK translation. Exposure to exosomes derived from sTNFR1-treated B cells also elevated TNF-α, IL-6, and CCL2 secretion in CDECs (**Figures 5D–F**). To confirm that the increase in tRNA-Pro-TGG was mediated by exosomal uptake, CDECs were pre-transfected with a tRNA-Pro-TGG antagomir before co-culture with exosomes from sTNFR1-stimulated B cells. The antagomir effectively reduced tRNA-Pro-TGG levels in B cells (**Supplementary Figure S4C**) and prevented the exosome-induced increase in tRNA-Pro-TGG in CDECs, thereby abolishing the upregulation of TNF-α, IL-6, and CCL2 expression (**Figures 5C–F**).

## Discussion

IgAN remains a leading cause of end-stage renal disease, characterized by the deposition of IgA in the glomeruli and progressive renal dysfunction (20, 21). Despite its prevalence, the molecular mechanisms underlying disease progression remain incompletely understood. In recent years, the potential of liquid biopsy to provide non-invasive biomarkers for the early detection and monitoring of IgAN has gained significant interest (22). Among the emerging biomarkers, tRNA-derived small RNAs (tsRNAs) have shown promise, given their regulatory roles in various cellular processes (5, 6). In this study, we provide novel evidence that exosomal tRNA-Pro-TGG, a tsRNA derived from tRNA-Pro, is significantly dysregulated in IgAN and serves as a potential biomarker for disease progression.

Our findings demonstrate that the levels of exosomal tRNA-Pro-TGG are markedly elevated in IgAN patients, particularly in those with progressive disease. This is consistent with previous reports suggesting that tsRNAs play a key role in the pathophysiology of IgAN, influencing inflammation and immune responses. Notably, the expression of tRNA-Pro-TGG was strongly correlated with serum soluble TNFR1 (sTNFR1) levels, a known marker of renal inflammation and disease severity. This correlation suggests that tRNA-Pro-TGG, potentially derived from activated lymphocytes, may contribute to the inflammatory processes driving disease progression in IgAN. The rationale for correlating tRNA-Pro-TGG specifically with sTNFR1 was based on its biological relevance and established clinical significance. Circulating sTNFR1 has been identified as an independent predictor of renal inflammation, tubular injury, and disease progression in IgAN and other chronic kidney diseases (17). In our cohort, sTNFR1 exhibited the strongest association with disease severity among several cytokines and clinical indices examined. Moreover, our experimental findings demonstrated that sTNFR1 regulates the release of tRNA-Pro-TGG enriched exosomes from B cells without affecting intracellular tRNA-Pro-TGG expression. These results suggest that sTNFR1 acts upstream of exosomal tRNA-Pro-TGG secretion and may participate in immune-mediated renal injury, providing a mechanistic basis for the observed correlation and supporting its selection as a relevant biomarker.

Exosomal tRNA-Pro-TGG is predominantly expressed in B cells, which are critical players in the immune response in IgAN. Our results further support the notion that sTNFR1 regulates the release of tRNA-Pro-TGG-enriched exosomes from B cells, without altering intracellular tRNA-Pro-TGG levels. This selective release mechanism could play a key role in the communication between immune cells and renal epithelial cells, facilitating the propagation of inflammatory signals to the kidney. Interestingly, we observed that exosomal tRNA-Pro-TGG significantly impacted the activation of collecting duct epithelial cells (CDECs), leading to increased levels of proinflammatory cytokines such as TNFα, IL-6, and CCL2. These findings underscore the potential role of tRNA-Pro-TGG in mediating renal inflammation and fibrosis, processes that are integral to the progression of IgAN.

Moreover, bioinformatic analyses identified several key signaling pathways, including the MAPK and PI3K-Akt pathways, as potential targets modulated by tRNA-Pro-TGG. Our experimental validation revealed that tRNA-Pro-TGG directly inhibits MAPK translation, leading to a reduction in MAPK protein expression. This suggests that tRNA-Pro-TGG may modulate key signaling cascades that govern cellular responses to injury and inflammation in IgAN. The dysregulation of MAPK signaling, in particular, may contribute to the altered cellular behavior observed in IgAN, including changes in inflammation.

The diagnostic potential of exosomal tRNA-Pro-TGG was also evaluated in our study. ROC curve analysis demonstrated that tRNA-Pro-TGG could effectively differentiate between progressive and nonprogressive IgAN, as well as between IgAN patients and healthy controls. These findings highlight the potential of tRNA-Pro-TGG as a non-invasive biomarker for early diagnosis and risk stratification in IgAN.

TNFR1 is present in the majority of cell types, including B lymphocytes, and serves as the principal receptor for soluble TNFα (sTNFα) (23). Activation of membrane-bound TNFR1 typically triggers canonical forward signaling pathways, which can result in cell death, survival, differentiation, or inflammatory responses (24). TNFR1 can also enter the circulation through two distinct mechanisms: proteolytic cleavage of the membrane-bound receptor by sheddases, producing a 27–30 kDa ectodomain, or incorporation into exosome-like vesicles as full-length 55 kDa receptors released by vascular endothelial cells, independent of the usual signaling complex. Initially, soluble TNFRs (sTNFRs) were believed to function exclusively as competitive inhibitors of sTNFα, reducing receptor engagement and mitigating TNFα-mediated inflammation (24). Subsequent studies, however, have revealed that sTNFR1 can serve as a ligand for membrane-bound TNFα, which then acts as a “receptor” to initiate reverse signaling. This process influences cell proliferation, cytokine production, oxidative responses, and immunoglobulin class switching, and has been reported to desensitize monocytes and macrophages to lipopolysaccharide stimulation. Furthermore, sTNFR1 can facilitate the presentation of sTNFα to membrane-associated TNFRs, thereby enhancing TNFα activity, especially when soluble TNFα levels are low. Independently of TNFα, sTNFR1 is also capable of inducing cellular activation, including the proliferation of endothelial cells. In the present study, we explored the relationship between sTNFR1 and tRNA-Pro-TGG in controlling exosome secretion from B cells and the resulting inflammatory responses in renal epithelial cells. Our *in vitro* experiments revealed several important findings. Treatment with sTNFR1 alone modulated the release of tRNA-Pro-TGG-enriched exosomes from B cells, without changing intracellular tRNA-Pro-TGG levels or directly provoking inflammation in renal epithelial cells. Specifically, B cells derived from patients with IgA nephropathy (IgAN) treated with recombinant sTNFR1 displayed unchanged intracellular tRNA-Pro-TGG, while the amount of tRNA-Pro-TGG within secreted exosomes was markedly increased. These results suggest that sTNFR1 specifically regulates the packaging of tRNA-Pro-TGG into exosomes rather than altering its intracellular production or stability. Exposure of collecting duct epithelial cells (CDECs) to sTNFR1 alone did not significantly enhance secretion of TNF-α, IL-6, or CCL2, indicating that sTNFR1 does not directly activate renal epithelial cells. Functional analyses demonstrated that sTNFR1 operates upstream of tRNA-Pro-TGG to promote exosome release, and these tRNA-Pro-TGG-enriched exosomes are essential for triggering proinflammatory responses in CDECs. Co-culture with exosomes derived from sTNFR1-treated B cells increased intracellular tRNA-Pro-TGG in CDECs and stimulated robust secretion of TNF-α, IL-6, and CCL2. Pre-transfection of CDECs with a tRNA-Pro-TGG antagomir before co-culture completely abolished both the elevation of tRNA-Pro-TGG and cytokine secretion, confirming the dependence on tRNA-Pro-TGG. Overall, our findings indicate that sTNFR1 controls the selective exosomal export of tRNA-Pro-TGG from B cells but does not directly activate renal epithelial cells. Through these tRNA-Pro-TGG-enriched exosomes, sTNFR1 indirectly induces proinflammatory signaling in CDECs, highlighting a sequential mechanism in which sTNFR1 acts upstream to regulate tRNA-Pro-TGG delivery, thereby mediating downstream renal inflammation.

In conclusion, our study provides compelling evidence that exosomal tRNA-Pro-TGG is a promising biomarker for IgAN progression and a key mediator of inflammation and immune response in this disease. Future studies will be needed to explore the precise mechanisms through which tRNA-Pro-TGG modulates immune cell interactions and renal inflammation. Additionally, the clinical utility of tRNA-Pro-TGG as a diagnostic and prognostic tool in IgAN warrants further validation in larger, multicenter cohorts.

## Data Availability

The datasets presented in this study can be found in online repositories. The names of the repository/repositories and accession number(s) can be found in the article/**Supplementary Material**.
