# Supplementary material for: B cell–derived exosomal tRNA-Pro-TGG served as a non-invasive biomarker and mediator of inflammation in progressive IgA nephropathy
Source: Front Immunol. 2025 Nov 21;16:1679290. doi: 10.3389/fimmu.2025.1679290 (PMC12678319; doi:10.3389/fimmu.2025.1679290)
Supplement: Supplementary file 1 [file DataSheet1.docx]

**Table s1. Patient demographics.**

|  |  | **Age, yr** | **Gender(Male,%)** | **eGFR, ml/min per 1.732** | **uPCR, g/g** | **Proteinuria (g/24h)** |
| --- | --- | --- | --- | --- | --- | --- |
| **RNA-sequencing** |  |  |  |  |  |  |
|  | **IgAN prog (10)** | 43±13 | 50 | 69.23±16.74 | 7.75±1.41 | 1.85±0.41 |
|  | **IgAN non prog (10)** | 42±10 | 50 | 82.24±17.19 | 2.41±1.03 | 0.45±0.22 |
|  | **HC(10)** | 44±13 | 50 | 91.76±12.84 | 0.11±0.12 | 0.05±0.02 |
| **qRT-PCR** |  |  |  |  |  |  |
|  | **IgAN prog (50)** | 42±14 | 50 | 70.86±15.59 | 8.37±1.96 | 1.00±0.42 |
|  | **IgAN non prog (50)** | 43±12 | 50 | 81.70±14.00 | 3.21±2.14 | 0.50±0.25 |
|  | **HC(50)** | 43±14 | 50 | 92.22±11.80 | 0.15±0.08 | 0.10±0.05 |

**Table s2. Sequences.**

|  |  | **Sequence** |
| --- | --- | --- |
| **tRNA-Pro-TGG** |  | GGCTCGTTGGTCTAGTGGTA |
| **tRNA-Pro-TGG** | **RT** | GTCGTATCCAGTGCAGGGTCCGAGGTATTCGCACTGGATACGACTCCCTG |
|  | **F** | GCGCGCGCGGGCCGGG |
| **U6** | **F** | CTCGCTTCGGCAGCACAT |
|  | **R** | TTTGCGTGTCATCCTTGCG |

**Table s3. The expression profile of tsRNA by RNA sequencing.**

**Table s4. The potential mRNA targets of differentially expressed tsRNAs between IgAN and HC predicted by TargetScan and miRDB.**

**Table s5. The potential mRNA targets of differentially expressed tsRNAs between progressive IgAN vs nonprogressive IgAN progressed predicted by TargetScan and miRDB.**


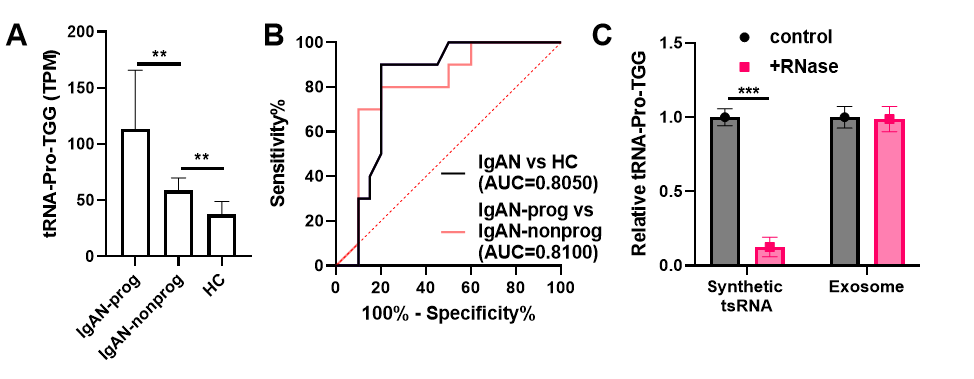


**Figure s1. TsRNAs contained in serum exosomes used as biomarkers for diagnosing IgAN. (A)** tRNA-Pro-TGG in serum exosomes from IgAN patients and Healthy controls. **(B)** ROC curve analysis of the tRNA-Pro-TGG in serum exosomes to distinguish IgAN from HC, and IgAN prog from IgAN non prog. **(C)** tRNA-Pro-TGG protected by exosome from RNase. *p* values are from unpaired Student’s t test or one-way ANOVA. ns: p > 0.05;**p < 0.01; ***p < 0.001.


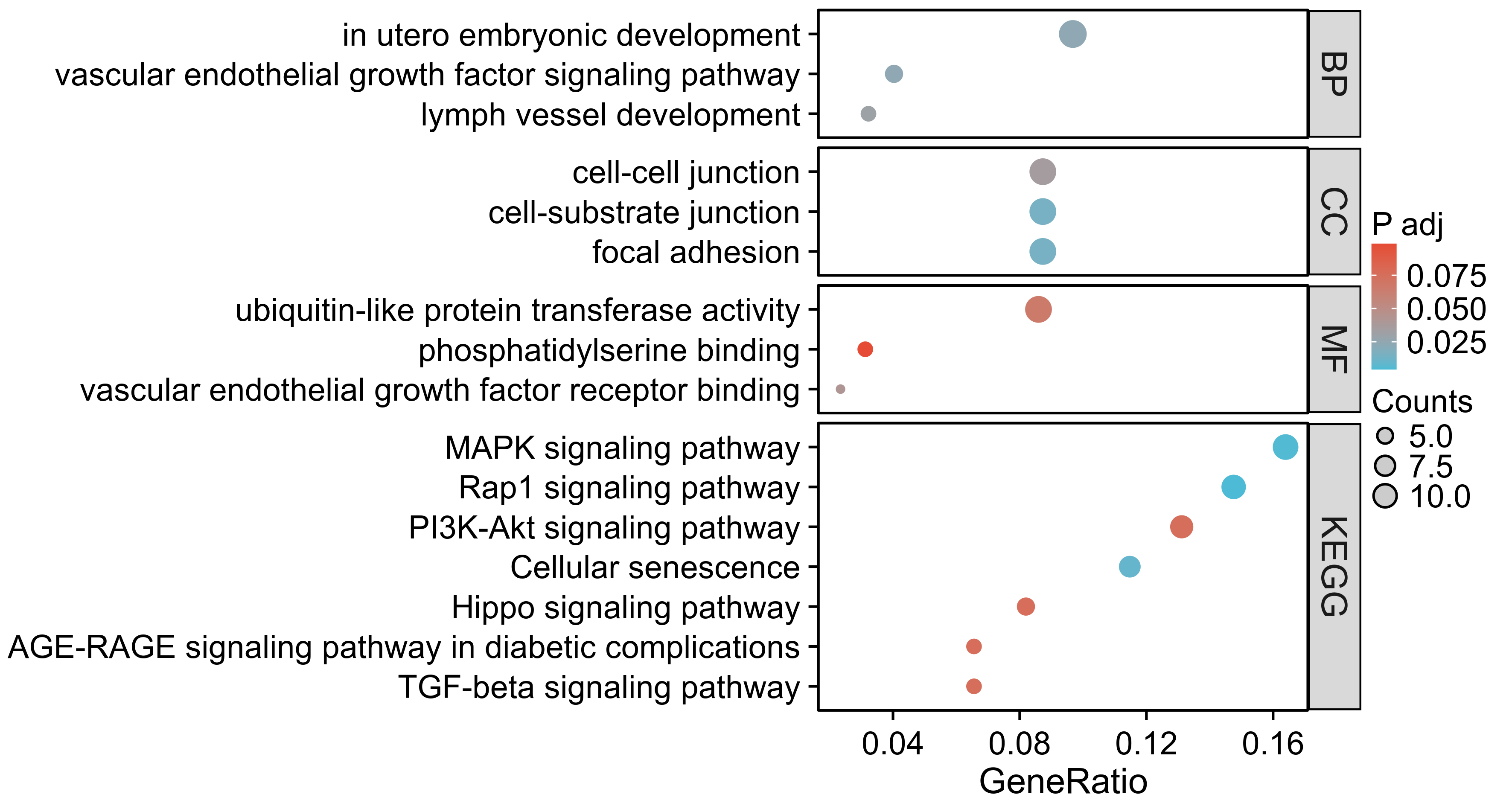


**Figure** **s2. GO and KEGG analyses of tRNA-Pro-TGG.**


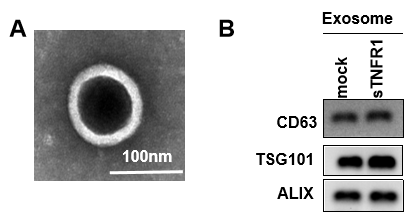


**Figure s3. Isolation and characterization of exosomes derived from B cells. (A)** TEM analysis of exosomes. The scale bar represents 100 nm. **(B)** Western blot analysis of exosomes derived from B cells treated with sTNFR1 (sTNFR1) or not (mock).

**
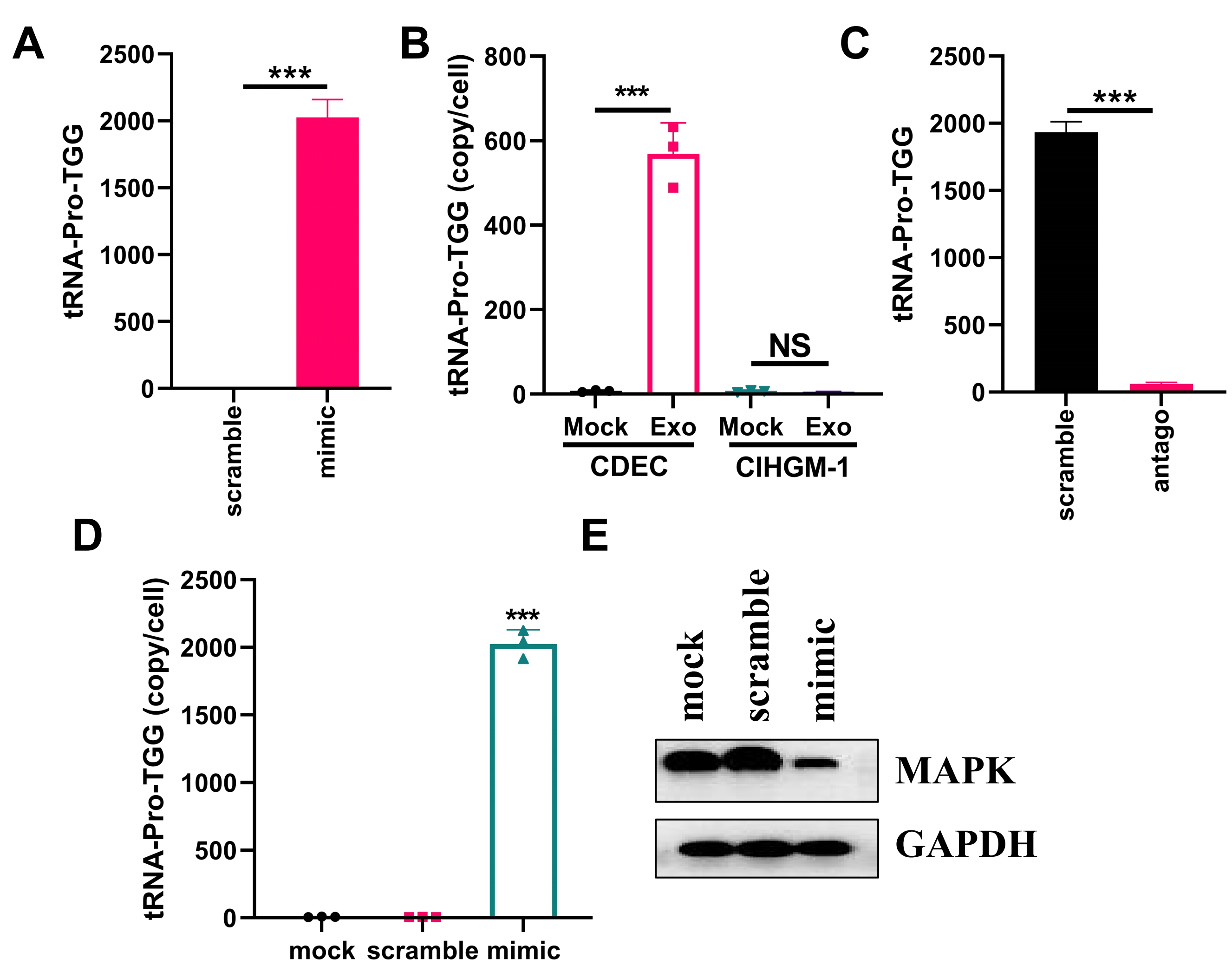
Figure s4. (A)** tRNA-Pro-TGG in the 293T cells transfected with tRNA-Pro-TGG mimic (mimic) or scramble RNA (scramble). **(B)** tRNA-Pro-TGG in the CDEC and CIHGM-1 cells co-cultured with Exosome derived from sTNFR1-exposed B cells (Exo) or not (Mock). **(C)** tRNA-Pro-TGG in the B cells transfected with tRNA-Pro-TGG antago (antago) or scramble RNA (scramble). **(D)** tRNA-Pro-TGG in the CDEC cells transfected with tRNA-Pro-TGG mimic (mimic) or scramble RNA (scramble). **(E)** MAPK in CDECs treated with scramble RNA or tRNA-Pro-TGG mimic. The data from three independent experiments (n = 3) are presented as the means ± SDs. *p* values are from unpaired Student’s t test or one-way ANOVA . ns: p > 0.05; ***p < 0.001.
